# Comparative Community Proteomics Demonstrates the Unexpected Importance of Actinobacterial Glycoside Hydrolase Family 12 Protein for Crystalline Cellulose Hydrolysis

**DOI:** 10.1128/mBio.01106-16

**Published:** 2016-08-23

**Authors:** Jennifer Hiras, Yu-Wei Wu, Kai Deng, Carrie D. Nicora, Joshua T. Aldrich, Dario Frey, Sebastian Kolinko, Errol W. Robinson, Jon M. Jacobs, Paul D. Adams, Trent R. Northen, Blake A. Simmons, Steven W. Singer

**Affiliations:** a Joint BioEnergy Institute, Emeryville, California, USA; b Biosciences Directorate, Lawrence Berkeley National Laboratory, Berkeley, California, USA; c Biological and Materials Science Center, Sandia National Laboratories, Livermore, California, USA; d Biological Sciences Division, Pacific Northwest National Laboratory, Richland, Washington, USA; e Environmental Molecular Sciences Laboratory, Pacific Northwest National Laboratory, Richland, Washington, USA; f Faculty of Biotechnology, University of Applied Sciences Mannheim, Mannheim, Germany

## Abstract

Glycoside hydrolases (GHs) are key enzymes in the depolymerization of plant-derived cellulose, a process central to the global carbon cycle and the conversion of plant biomass to fuels and chemicals. A limited number of GH families hydrolyze crystalline cellulose, often by a processive mechanism along the cellulose chain. During cultivation of thermophilic cellulolytic microbial communities, substantial differences were observed in the crystalline cellulose saccharification activities of supernatants recovered from divergent lineages. Comparative community proteomics identified a set of cellulases from a population closely related to actinobacterium *Thermobispora bispora* that were highly abundant in the most active consortium. Among the cellulases from *T. bispora*, the abundance of a GH family 12 (GH12) protein correlated most closely with the changes in crystalline cellulose hydrolysis activity. This result was surprising since GH12 proteins have been predominantly characterized as enzymes active on soluble polysaccharide substrates. Heterologous expression and biochemical characterization of the suite of *T. bispora* hydrolytic cellulases confirmed that the GH12 protein possessed the highest activity on multiple crystalline cellulose substrates and demonstrated that it hydrolyzes cellulose chains by a predominantly random mechanism. This work suggests that the role of GH12 proteins in crystalline cellulose hydrolysis by cellulolytic microbes should be reconsidered.

## IMPORTANCE

Cellulose is the most abundant organic polymer on earth, and its enzymatic hydrolysis is a key reaction in the global carbon cycle and the conversion of plant biomass to biofuels. The glycoside hydrolases that depolymerize crystalline cellulose have been primarily characterized from isolates. In this study, we demonstrate that adapting microbial consortia from compost to grow on crystalline cellulose generated communities whose soluble enzymes exhibit differential abilities to hydrolyze crystalline cellulose. Comparative proteomics of these communities identified a protein of glycoside hydrolase family 12 (GH12), a family of proteins previously observed to primarily hydrolyze soluble substrates, as a candidate that accounted for some of the differences in hydrolytic activities. Heterologous expression confirmed that the GH12 protein identified by proteomics was active on crystalline cellulose and hydrolyzed cellulose by a random mechanism, in contrast to most cellulases that act on the crystalline polymer in a processive mechanism.

## INTRODUCTION

The enzymatic hydrolysis of cellulose is a critical activity in the natural cycling of carbon and the biochemical conversion of plant biomass to fuels and chemicals (1). Plant-derived cellulose is often in crystalline form, which is recalcitrant to hydrolysis (2). Microorganisms, predominantly filamentous fungi and bacteria, have evolved families of glycoside hydrolases that are able to hydrolyze crystalline cellulose. Exoglucanases, predominantly from glycoside hydrolase families 7 and 48 (GH7 and GH48, respectively), produce cellobiose at the reducing end of the cellulose chain (3). Exoglucanases from the GH6 family primarily produce cellobiose from the nonreducing end of the chain. Processive endoglucanases, predominantly from GH families 5, 6, and 9, also hydrolyze crystalline cellulose to produce cellobiose (4). A ubiquitous feature of both the exoglucanases and processive endoglucanases is the presence of a carbohydrate-binding domain linked to the catalytic domain, which has been shown to improve the efficiency of the catalytic domain (5). Model cellulolytic organisms with free enzymes have different complements of these enzymes. For example, *Trichoderma reesei* (syn. *Hypocrea jecorina*) expresses GH7 and GH6 exoglucanases for crystalline cellulose hydrolysis (6), while *Thermobifida fusca*, a thermophilic actinobacterium, produces a processive endoglucanase (GH9) and exoglucanases (GH6 and GH48) that function synergistically to hydrolyze crystalline cellulose (7). Cellulolytic bacteria from the *Caldicellulosiruptor* genus produce large multidomain glycoside hydrolases, such as CelA, which combines two catalytic domains (GH9 and GH48) that function in tandem to hydrolyze crystalline cellulose (8).

A number of other GH families are capable of hydrolyzing cellulosic substrates, although these have mostly been demonstrated on soluble substrates. Among the most enigmatic is the GH12 family, which is widely distributed in bacteria and fungi. GH12 proteins have been characterized as endoglucanases, xyloglucanases, and xylanases and have a structural resemblance to proteins in the GH11 family, which are predominately xylanases (9). The *T. reesei* GH12 protein has been characterized as an endoglucanase and is dispensable for the hydrolysis of crystalline cellulose (10). Fungal GH12 proteins lack cellulose binding modules (CBMs), suggesting that they cannot bind to the cellulose chain (11). Bacterial GH12 proteins characterized as endoglucanses from *Rhodothermus marinus* and *Thermotoga maritima* have been reported to have limited hydrolytic activity on insoluble cellulose substrates (12, 13). Recently, a GH12 protein from the actinobacterium *Acidothermus cellulolyticus* was characterized, and in contrast to the other characterized bacterial GH12 proteins, demonstrated significant activity on insoluble cellulose substrates, including Avicel (14). This observation and the prevalence of GH12 genes in the genomes of cellulolytic *Actinobacteria* suggested that these proteins may have a greater role in the hydrolysis of crystalline cellulose than previously understood (15). The biotechnological and ecological relevance of actinobacterial cellulases is highlighted by the demonstration that mixtures of cellulases from *Actinobacteria* perform comparably to commercial fungal cellulases (16) and the importance of *Actinobacteria* in environments with high rates of cellulose depolymerization like compost (17).

*Thermobispora bispora* (syn. *Microspora bispora*) is an obligate thermophilic actinobacterium from the suborder *Streptosporangineae* (18). The genome of the *T. bispora* type strain (ATCC 43833) contains a number of cellulase genes (coding for the GH48, GH6_endoglucanase, GH6_exoglucanase, and GH12 proteins); however, its ability to grow on cellulosic substrates appears to be limited (15, 19). In contrast, another *T. bispora* strain, NRRL 15568, which was identified by morphology and which has not been genomically sequenced, was reported to readily grow on cellulosic substrates at 60°C and produce both exoglucanases and endoglucanases (20, 21). Here we report that the cultivation of thermophilic cellulolytic microbial communities adapted from compost produced a consortium dominated by a population closely related to the *T. bispora* type strain. The unexpected importance of the *T. bispora* GH12 protein for hydrolysis of crystalline cellulose was demonstrated by combining comparative community proteomics and biochemical measurements.

## RESULTS

### Divergent cellulase activities in culture supernatants.

**FIG 1:**
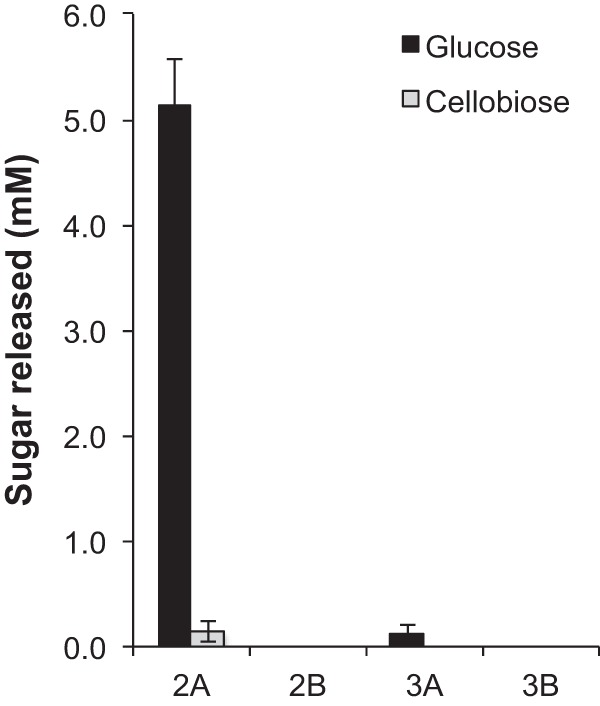
Saccharification of microcrystalline cellulose (MCC) with MCC-adapted culture supernatants at 60°C after 72 h. Supernatants were collected from each culture and incubated with 2% (wt/vol) Avicel. Enzyme loading was set at 5 mg enzyme/g glucan as measured by the BCA assay. Glucose and cellobiose were measured by HPLC. Error bars represent standard deviations from the mean from three technical replicates.

**FIG 2:**
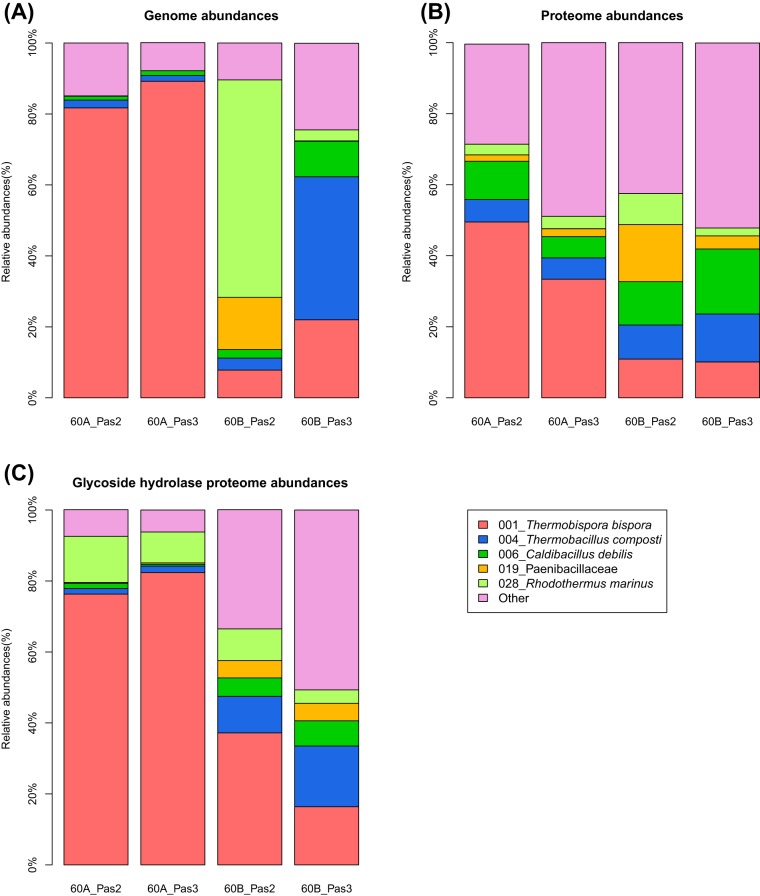
(A) Relative metagenomic, (B) metaproteomic abundances, and (C) glycoside hydrolase metaproteomic abundances of the adapted communities. The metagenomic and metaproteomic abundances were normalized to 100% for each sample. Different colors indicate different draft genomes obtained from binning of the metagenomic assembly. For clarity, only draft genomes with at least 10% relative abundance in any of the metagenomic samples are displayed; all other draft genomes are summarized by “Other.”

Duplicate cellulolytic cultures of green waste compost communities were cultivated to test the reproducibility of community formation compared to previous adaptation experiments (22). In contrast to previous replicated adaptation experiments, measurement of cellulase activities during early development of these enrichments demonstrated large differences between culture lineages. One lineage (passages 2A and 3A) had hydrolytic activities on carboxymethyl cellulose (CMC) and *p*-nitrophenyl-β-d-cellobioside (pNPC) activities that were 4- to 20-fold higher than those of the second lineage (passages 2B and 3B) (Table 1); in contrast, activities on *p*-nitrophenyl-β-d-glucanopyranoside (pNPG) were higher in the 2B and 3B supernatants. Saccharification of crystalline cellulose with culture supernatants indicated that the supernatant from one consortium (2A), possessed 20-fold greater activity than the succeeding culture in the lineage (3A), and supernatants from the B lineage had no measurable saccharification activity (Fig. 1). Metagenomic sequencing was performed to determine the community composition and genomic potential of the community members. Coassembly and binning of assembled contigs from the four metagenomic data sets indicated that the A lineage was dominated (>80% abundance) by a population closely related to cellulolytic actinobacterium *Thermobispora bispora* (>99% amino acid identity) (Fig. 2A; see Tables S1 and S2 in the supplemental material). The B lineage had variable community structures with abundant populations closely related to *Rhodothermus marinus* and members of the *Firmicutes* (*Thermobacillus composti* and *Caldibacillus debilis*). Comparison of the population genome of *T. bispora* recovered from the cellulose cultivations with the genome of *T. bispora* ATCC 43833 indicated that the two strains were very similar: the average number of mismatches is 1 bp, and the average number of indels is 0.01 bp per gene.

**TABLE 1:** Cellulase and hemicellulase activities recovered from thermophilic bacterial communities adapted to microcrystalline cellulose

Passage	Sp act (U/mg) on substrate^a^:		
	CMC^b^	pNPC^c^	pNPG
2A	1.26 ± 0.29	466.0 ± 92.7	57.6 ± 4.1
2B	0.29 ± 1.42	23.2 ± 0.7	114.0 ± 9.2
3A	1.42 ± 0.06	502.1 ± 5.1	21.0 ± 1.0
3B	0.37 ± 0.02	36.0 ± 1.9	457.5 ± 0.4

a Bacterial supernatants were assayed with the substrates CMC, pNPG, and pNPC. Specific activities are based on protein concentrations obtained using the BCA method. Three replicates were performed, and the ranges represent the standard deviation from the mean. The activities represented by each substrate are described in Materials and Methods.

b For CMC, 1 U represents 1 μmol sugar released min^−1^ ml^−1^ of supernatant.

c For pNPC, 1 U represents 1 μmol *p*-nitrophenol released min^−1^ mg^−1^ protein.

### Comparative supernatant proteomics.

**FIG 3:**
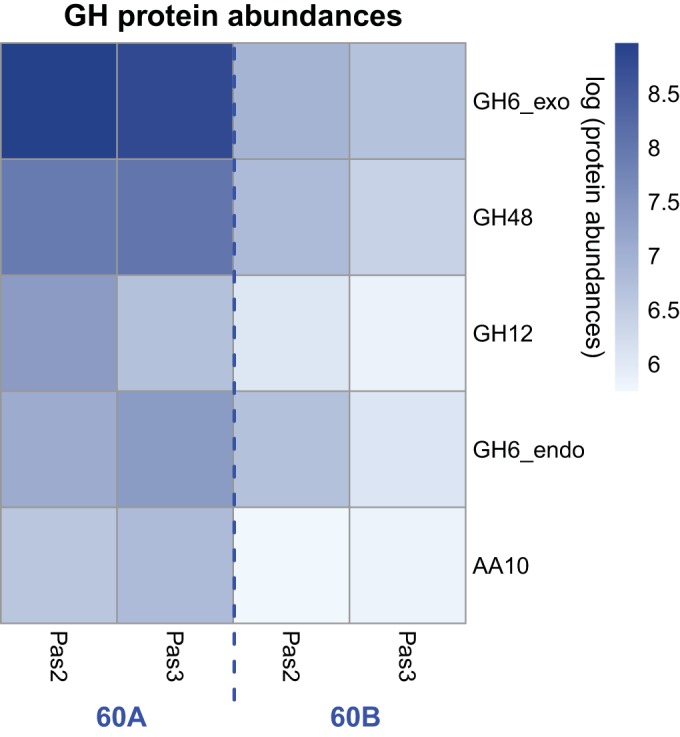
Heat map representing the proteomic abundances of the *T. bispora* cellulases (the GH12, GH6_exo, GH48, GH6_endo, and AA10 proteins) as measured by LC-MS/MS-based iTRAQ quantification. Lighter colors indicate lower log proteomic abundances of each cellulase in four different samples, and darker colors indicate higher log abundances. Pas2 and Pas3 represent supernatants recovered from passage 2 and passage 3 of each culture lineage, respectively.

Comparative mass spectrometry-based proteomic analysis of the four supernatants using iTRAQ quantification was performed to identify the proteins responsible for the large differences in saccharification activity between the supernatants. For lineage A (cultures 2A and 3A), *T. bispora* proteins were the most abundant proteins (49.4% in 2A and 33.1% in 3A) (Fig. 2B; see Table S3 in the supplemental material). In lineage B, however, the abundances of *T. bispora* proteins were reduced to 10.7% and 10.0% in 2B and 3B, respectively. Most of the glycoside hydrolases (GH) present in 2A and 3A were produced by *T. bispora*—the total proteomic GH abundances of 2A and 3A were several-fold larger than those of 2B and 3B (see Table S4 in the supplemental material), and the relative proteomic abundances of 2A and 3A also demonstrated that the *T. bispora* GH proteins accounted for 76% and 82% of the total GH proteome (Fig. 2C; see Table S4). Of these *T. bispora* GH proteins, cellulases such as an exoglucanase from glycoside hydrolase family 6 (GH06_exo), an endoglucanase from GH family 6 (GH06_endo), a cellobiohydrolase from GH family 48 (GH48 protein), a lytic polysaccharide monooxygenase of the auxiliary activity family 10 (AA10), and a poorly characterized enzyme from GH family 12 (GH12 protein) were detected in all supernatants (Fig. 3). Each of these proteins contained a catalytic domain linked to a family 2 cellulose binding module (CBM2). All of the five cellulases were more abundant in the A lineage than in the B lineage (Fig. 3). The most abundant protein in lineage A was GH6_exo, followed by the GH48, GH12, and GH6_endo proteins.

To identify which protein(s) contributed to the difference in glucose release between the supernatants, Pearson’s correlation coefficients were estimated for the glucose release and the protein abundances of the five cellulases from *T. bispora* (see Table S5 in the supplemental material). Among all five cellulases, only the GH12 protein is significantly correlated with the produced amount of glucose (*P* = 0.01), suggesting that it may be the most important factor of these proteins to the relatively large amount of glucose production in 2A. The correlation between the peptide abundances of all proteins from the 30 genome bins and the glucose production amount was also estimated. In total, 117 proteins (including the detected GH12 protein shown in Fig. 3) were significantly correlated (*P* ≤ 0.05) with the amount of produced glucose (see Fig. S1 in the supplemental material). Only 2 out of the 117 proteins were glycoside hydrolases, and the GH12 protein is 10 times more abundant than the other glycoside hydrolase, which was a *Caldibacillus debilis* GH1 protein (bin006) that was annotated as a β-glucosidase. The proteomics data suggested that the higher abundance of the *T. bispora* GH12 protein in the 2A supernatant compared to the other supernatants may have contributed to its high relative activity in crystalline cellulose hydrolysis.

### Expression and characterization of *T. bispora* cellulases.

**FIG 4:**
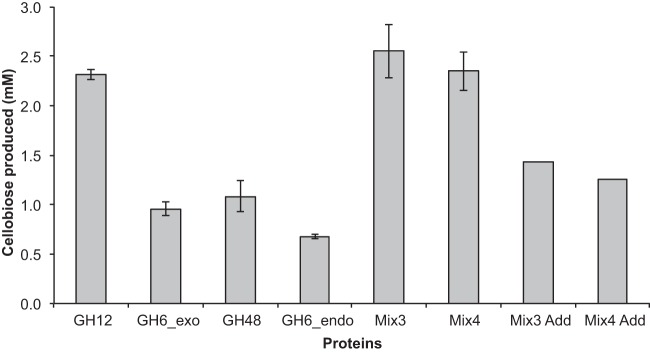
Production of cellobiose from Avicel by *T. bispora* cellulases expressed in *E. coli*. Mix3 (GH12, GH6_exo, and GH48) and Mix4 (GH12, GH6_exo, GH48, and GH6_endo) represent samples mixed at equal concentrations of each protein. Mix3 Add and Mix4 Add represent sums of individual activities of each protein at the specified concentration. Proteins were purified and saccharifications were performed as described in Materials and Methods. Glucose and cellobiose were measured by HPLC, although only cellobiose is reported in the figure. Measured glucose release is depicted in Fig. S2 in the supplemental material.

To test the activity of the GH12 protein in comparison to those of the other *T. bispora* hydrolytic cellulases, the abundant *T. bispora* cellulases (GH6_exo, GH12, GH48, and GH6_endo proteins) identified in the proteomics experiment were expressed in *Escherichia coli* as 8×His proteins and purified by Ni affinity chromatography. The GH12 protein exhibited higher activity than the other cellulases on crystalline cellulose, with the GH12 protein possessing ~2.5-fold-higher levels of activity in the release of cellobiose from Avicel in comparison to the GH6_exo and the GH48 proteins (Fig. 4; see Fig. S2 in the supplemental material). Mixtures of these proteins (GH12, GH6_exo, and GH48 proteins and GH12, GH6_exo, GH48, and GH6_endo proteins) demonstrated synergistic activity, exhibiting ~2-fold levels of cellobiose release from Avicel compared to adding the activity of each protein individually.

**FIG 5:**
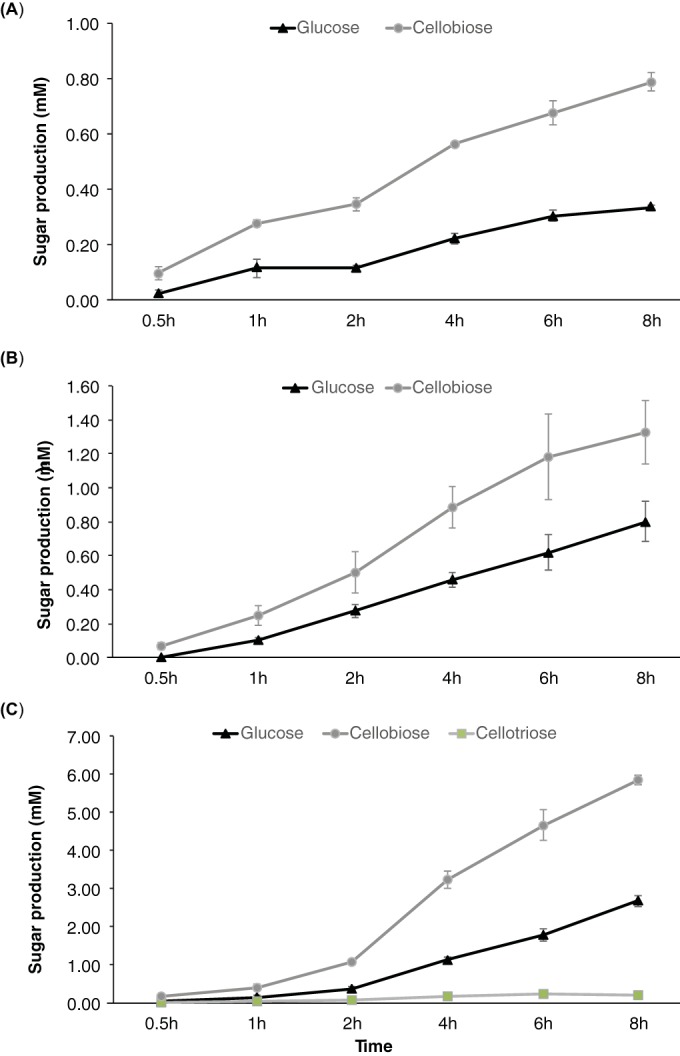
Time course experiments for GH12 protein hydrolysis of Avicel (A), filter paper (B), and PASC (C). Sugar products were derivatized with oxime and measured by NIMS. Each point represents the average of three independent replicates.

Product distributions of the cellulose hydrolysis were compared for the GH12, GH6_exo, and GH48 proteins, the most active cellulases on Avicel, using nanostructure-initiator mass spectrometry (NIMS) by posthydrolysis tagging of sugar products using oxime chemistry (23). This technique has provided rapid analysis of the products of glycoside hydrolases, allowing multiplexed comparisons of polysaccharide hydrolysis by glycoside hydrolases (24). Initial rates and products of hydrolysis were measured, and phosphoric acid-swollen cellulose (PASC) and filter paper were added to broaden the substrate profile of these glycoside hydrolases. The NIMS results demonstrated that initial hydrolysis by GH12 protein of the cellulose substrates after 8 h produced cellobiose and glucose in an ~2:1 ratio (Fig. 5). An independent experiment indicated that the GH12 protein rapidly hydrolyzed cellotriose (~50% hydrolysis in 1 h), consistent with the observed cellobiose/glucose ratio. Cellulose hydrolysis by the GH6_exo and GH48 proteins predominantly produced cellobiose, with glucose and cellotriose observed as minor products, suggesting that they function as cellobiohydrolases (see Fig. S3 and S4 in the supplemental material). In contrast, the NIMS result suggested that the GH12 protein hydrolyzed the insoluble substrates by a random mechanism and was not a processive enzyme. This observation was supported by a processivity assay that compared the ratio of the soluble and insoluble reducing ends in filter paper hydrolysis by the GH12 protein. The ratio of soluble to insoluble reducing ends was 56:44, which corresponded to a processivity ratio of 1.3.

## DISCUSSION

This study demonstrates that linking enzymatic assays with comparative community proteomics of related microbial consortia can uncover new activities, even of community members closely related to cultivated bacteria like *Thermobispora bispora*. Most comparative community proteomic studies have emphasized the differential presence of proteins when comparing cultivation and environmental conditions (25, 26), while this study adds comparative activity measurements of the proteins produced by consortia as well as heterologous expression of targeted proteins to test the hypotheses generated by the differential proteomics analysis. Though it is likely that multiple proteins contributed to the increased saccharification activity in the 2A supernatant, the importance of the *T. bispora* GH12 family for crystalline cellulose hydrolysis was highlighted by comparative proteomics and supported by biochemical measurements of the individual proteins.

The genesis of this study was to test the reproducibility of community adaptation to growth on microcrystalline cellulose. A previous set of adaptation experiments on microcrystalline cellulose and wheat arabinoxylan demonstrated that community structures were largely reproduced after two passages of the communities inoculated with a compost sample (22). These community structures were mostly made of members of the *Firmicutes* and *Bacteroidetes*, and this structure was also observed in the B lineage of the adaptions in this study. All of these communities had relatively low levels of cellulase activities, as measured by the 3,5-dinitrosalicylic acid (DNS)-based endoglucanase activities with carboxymethyl cellulose as the substrate. In all adaptations, a population closely related to *Thermobispora bispora* was present at low abundance (<5%). In contrast, the *T. bispora* population was >80% in the two passages in the A lineage, and its glycoside hydrolase enzymes were the most abundant GH proteins as measured by proteomics. Therefore, community formation appears not to be reproducible, and different community structures may arise from the same inoculum. This observation is consistent with the existence of founder effects in the formation of microbial communities that may affect their overall structure (27).

The behavior of the *T. bispora* population in the A lineage of the cellulolytic consortia resembles the *T. bispora* strain described by Eveleigh (NRRL 15568), which grew at 60°C and secreted large amounts of exoglucanases and endoglucanases (20). Attempts to obtain an isolate from the *T. bispora* population from the A lineage were not successful, as were attempts to revive a freeze-dried culture of NRRL 15568. The behavior of the *T. bispora* A lineage population is not consistent with the *T. bispora* type strain (ATCC 43833), as this strain has an optimal growth temperature at 45°C and high levels of cellulases are not produced during cultivation on microcrystalline cellulose (MCC) (15, 19). Comparison of the population genome of the *T. bispora* from the A lineage with the genome of the *T. bispora* type strain indicated that they were >99% identical at the amino acid level. These results suggests that even though the genomes of the different strains of *T. bispora* are very similar, subtle changes in genomic content and cultivation conditions may be responsible for high levels of cellulase secretion by *T. bispora* growing at 60°C.

While *Actinobacteria* are found in environments with high rates of biomass deconstruction and broadly possess the metabolic potential for crystalline cellulose hydrolysis, detailed biochemical and systems biological studies of cellulose hydrolysis have only been performed on a few model systems. The most comprehensively studied cellulolytic actinobacterium is *Thermobifida fusca*, for which detailed biochemical studies have demonstrated that the most important enzymes for crystalline cellulose hydrolysis are a processive GH9 endoglucanase and two exocellulases, the GH48 and GH6 proteins. The GH9 and GH48 enzymes act in synergy to hydrolyze the reducing ends of the cellulase chain, while the GH6 enzyme hydrolyzes the nonreducing ends (7). *T. fusca* has no GH12 protein encoded in its genome. Transcriptomic and proteomic measurements of the response of *Streptomyces* sp. strain Sirex AA-E, isolated from a pine-boring wood wasp, to crystalline cellulose demonstrated that a GH6 protein and GH48 protein were the most abundant proteins secreted (16). Additionally, the third most abundant protein was a lytic polysaccharide monooxygenase (AA10), an oxidative enzyme that has been demonstrated to work in synergy with the glycoside hydrolases to depolymerize crystalline cellulose. *T. fusca* also has a highly expressed AA10 protein that may have enhanced the hydrolytic activity of the GH9, GH48, and GH6 proteins on crystalline cellulose. The GH12 protein was not prominent in the *Streptomyces* sp. strain Sirex AA-E secretome; however, a related cellulolytic *Streptomyces* strain, DpondAA-B6, possessed a GH12 protein that was 6.2% of the total extracellular proteome of the DpondAA-B6 strain grown on crystalline cellulose (28). An extensive survey of sequenced *Streptomyces* species demonstrated that GH12 proteins were present in the genomes of all of the members of cellulolytic clades, suggesting they play an essential role in cellulose depolymerization (29). *Acidothermus cellulolyticus*, a thermophilic actinobacterium, produced multiple thermostable endoglucanases and β-glucosidases, of which the *A. cellulolyticus* GH5 protein (E1) is the most comprehensively studied (30, 31). Genomic analysis of *A. cellulolyticus* demonstrated that the majority of the cellulase genes were in a gene cluster that contained genes coding for GH5, GH6, GH12, GH48, and GH74 proteins (32). Many of these *A. cellulolyticus* cellulases contain multiple carbohydrate-binding modules of families 2 and 3 (CBM2 and -3, respectively), and the cluster contains two GH12 catalytic domains: one domain with a CBM2 domain, similar to the *T. bispora* protein, and a second domain found in a multidomain protein with a GH6 protein and two CBMs. The precise roles of the *A. cellulolyticus* cellulases in cellulose hydrolysis have not been completely elucidated. The work with the *T. bispora* cellulases described here and the characterization of the *A. cellulolyticus* GH12 CBM2 described in the introduction suggest that the GH12 catalytic domains may have a larger role in crystalline cellulose hydrolysis than has been envisioned.

The observation of random hydrolytic activity by the *T. bispora* GH12 protein is consistent with a mechanism where the GH12 protein randomly cleaves cellulose chains in the crystalline material and the GH48 and GH6 cellobiohydrolases cleave the exposed cellulose chains at the reducing and nonreducing ends, which may account for the synergistic increase in activity when these GH proteins are mixed. The abundant AA10 protein may oxidize highly crystalline cellulose regions, facilitating hydrolysis by the GH proteins (33). This model is different from the classical endoglucanase-exoglucanase synergy model, in which the endoglucanases hydrolyzed amorphous regions, which exposes crystalline regions for hydrolysis by exoglucanases (34, 35). These results suggest that the GH12 protein may have an underappreciated role in the crystalline cellulose hydrolysis, and more comprehensive biochemical and systems biological studies of cellulolytic *Actinobacteria* may further refine its role and importance.

## MATERIALS AND METHODS

### Sample collection and enrichment of thermophilic consortia.

Compost samples were collected from a free municipal green waste composting program in Berkeley, CA (37°52′08.0″N 122°18′46.1″W), referred to here as Berkeley Green Waste (BGW) compost. The green waste consisted of yard trimmings and discarded food waste from an end-stage compost pile. Samples were transported to the lab at room temperature and stored at 4°C until inoculation. The adaptation of thermophilic consortia to purified substrates was described previously (36). Briefly, microcrystalline cellulose (0.5% wt/vol; Sigma, St. Louis, MO) was the sole supplemented carbon and energy source in 50 ml of M9 medium augmented with vitamins and buffered with 10 mM 2-(*N*-morpholino)ethanesulfonic acid (MES) at a final pH of 6.5 (37). Approximately 0.5 g of the BGW compost material was inoculated into the initial enrichments. Two biological replicates, referred to as A and B, were incubated in parallel at 60°C under aerobic conditions at 200 rpm. The enrichments were serially passed through three sets of liquid cultures (10% [vol/vol] inoculum), referred to as passages.

### Measurement of protein concentration and glycoside hydrolase activity.

At the end of each serial passage, DNA was isolated from cell pellets with the FastDNA spin kit for soil and the FastPrep instrument (MP Biomedicals, Santa Ana, CA). Protein concentrations were determined by bicinchoninic (BCA) assay (Pierce BCA protein assay kit; Thermo Scientific, Rockford, IL) methods, using a 96-well plate (200-µl reaction volume) with bovine serum albumin as the standard. Cellulase activity assays were conducted as previously described (38). Briefly, endoglucanase and xylanase activities were assessed by the 3,5-dinitrosalicylic acid (DNS) method, using carboxymethyl cellulose and birchwood xylan as the substrates, respectively, with either glucose or xylose as the standard (39). The enzyme reaction volume was 80 µl followed by 80 µl of DNS solution to measure released reducing sugars. One unit of cellulase or xylanase activity was defined as the amount of crude protein releasing a micromole of reducing sugar per minute per milliliter of supernatant volume. Cellobiohydrolase (pNPC), β-d-glucosidase (pNPG), and β-d-xylosidase (pNPX) activities were determined using their respective *p*-nitrophenyl sugar substrates. The *p*-nitrophenyl substrate (90 µl) was incubated with 10 µl of diluted enzyme, incubated for 30 min, and quenched with 50 µl of 2% cold sodium bicarbonate. The absorbance of released *p*-nitrophenol was measured at 410 nm. Activities using *p*-nitrophenyl substrates were calculated as micromoles of *p*-nitrophenol released per minute per milligram of crude protein.

### Analysis of metagenomic data sets.

Sequencing of the metagenomes was carried out by the Joint Genome Institute as previously described (40). The sequencing reads of four metagenomic data sets, including lineage A passage (2A), lineage A passage 3 (3A), lineage B passage 2 (2B), and lineage B passage 3 (3B), were coassembled using SPAdes 3.6 (41). The coassembled scaffolds were then binned using MaxBin 2.0 (40, 42). A customized Perl script was implemented to remove redundant genomic regions in the binned genomes. Briefly, this script will first self-BLAST each of the binned genomes using BLASTN, detecting distinct regions that were overlapped by >1,000 bp with identity of >95% and leave only one copy of the overlapped regions on the longest scaffold. To analyze the genetic content of the binned genome that was most closely related to *Thermobispora bispora*, genes were predicted from the recovered genome using Prodigal (43) and compared against the *T. bispora* DSM 43833 genome downloaded from NCBI (accession ID NC_014165.1) using BLASTX with an E value set to 1e−5 and max_target_seqs set to 1. Glycoside hydrolase (GH) genes were predicted by running HMMER3 (44) on the HMM files downloaded from the dbCAN server (45).

### Saccharification with crude supernatants on cellulose.

Saccharifications were performed in the presence of 2% (wt/vol) Avicel (Sigma, St. Louis, MO). Each mixture was prepared in 50 mM MES (pH 6.0) with 5 mg protein from crude supernatants per g glucan in biomass to a final volume of 5 ml in a 15-ml Falcon tube. Saccharifications were carried out 60°C in a shaker for 72 h. All hydrolysates were collected via centrifugation at 21,000 × *g* for 5 min and 0.45-µm-pore filtered to remove large biomass particles prior to sugar analysis. After filtration, samples were kept frozen at −20°C and thawed prior to analysis. Glucose concentrations were measured on an Agilent 1200 series high-performance liquid chromatography (HPLC) system equipped with an Aminex HPX-87H column (Bio-Rad, Hercules, CA) and refractive index detector. Samples were run with an isocratic 4 mM sulfuric acid mobile phase. Sugar concentrations were determined using standards containing both glucose and cellobiose.

### Comparative proteomic analysis of thermophilic bacterial enrichment supernatants.

Each supernatant was thawed and buffer exchanged through a 3,000 molecular weight cutoff (MWCO) spin column (Millipore, Temecula, CA) into 100 mM NH_4_HCO_3_ (pH 8). Each sample was then transferred into a fresh, labeled centrifuge tube, and a bicinchoninic acid assay (BCA assay) (Thermo Scientific, Rockford, IL) was performed to determine the protein concentration. Powdered urea was added to the supernatant at a concentration of 8 M along with 10 mM dithiothreitol (DTT). The samples were sonicated and incubated at 60°C for 30 min with constant shaking at 800 rpm. Samples were then diluted 8-fold for preparation for digestion with 100 mM NH_4_HCO_3_–1 mM CaCl_2_, and sequencing-grade modified porcine trypsin (Promega, Madison, WI) was added to all protein samples at a 1:50 (wt/wt) trypsin/protein ratio for 3 h at 37°C. Digested samples were desalted using a 4-probe positive-pressure Gilson GX-274 ASPEC system (Gilson, Inc., Middleton, WI) with Discovery C_18_ 100-mg/ml solid-phase extraction tubes (Supelco, St. Louis, MO) as previously described (46). The samples were concentrated down to ~30 µl using a Speed Vac, and a final BCA assay was performed to determine the peptide concentration.

The samples were measured and vialed to contain 50 µg each, and the volumes were brought up to 15 µl using 0.5 M tetraethylammonium bromide (TEAB; Sigma, St. Louis, MO) in a low-protein-binding 1.5-ml centrifuge tube. The pH of each sample was measured and brought to over pH 8 using 1 M TEAB. Each vial of 8-plex iTRAQ reagent (AB Sciex, Framingham, MA) was brought to room temperature. The reagents were pulse spun to ensure the contents were collected at the bottom, and 60 µl of isopropanol was added to each reagent vial. The reagents were thoroughly vortexed, spun down, and added to the appropriate sample. Only 4 out of the 8-plex iTRAQ channels were utilized for the current comparison, which included reporter ions 115, 117, 119, and 121, corresponding to supernatant sample passages 2B, 3B, 3A, and 2A, respectively. Samples were vortexed and spun down to incubate at room temperature for 2 h, at which time 100 µl of Nanopure water was added to hydrolyze the sample and incubated for an additional 30 min. The samples were partially dried down in a Speed Vac to remove the organic solvent and then pooled to obtain 1 sample containing all iTRAQ-labeled preparations, followed by C_18_ extraction (as described above) and BCA assay to determine the final peptide mass for HPLC fractionation.

The sample was diluted to a volume of 900 µl with 10 mM ammonium formate buffer (pH 10.0), and resolved on an XBridge C_18_ column (250 by 4.6 mm, 5 µM, with 4.6- by 20-mm guard column) (Waters, Milford, MA). Separations were performed at 0.5 ml/min using an Agilent 1100 series HPLC system (Agilent Technologies, Santa Clara, CA) as previously described (47). Each 24th fraction was combined for a total of 24 samples (each with *n =* 4 fractions pooled), each with 50% acetonitrile rinsing. The fractions were then completely dried down, and 25 µl of 25 mM ammonium bicarbonate was added to each fraction for storage at −20°C until liquid chromatography-tandem mass spectrometry (LC-MS/MS) analysis.

All iTRAQ-labeled fractions were analyzed by LC-MS/MS with the LC component utilizing an automated 65-cm by 75-mm-inside-diameter (i.d.) reversed-phase capillary column, HPLC system, and PAL autosampler as previously described (47). The MS component consisted of a Thermo Scientific LTQ-Orbitrap Velos mass spectrometer (Thermo Scientific, San Jose, CA) operated with settings previously described (47)

LC-MS/MS raw data were converted into dta files using Bioworks Cluster 3.2 (Thermo, Fisher Scientific, Cambridge, MA), and the MSGF+ algorithm (48) was used to search MS/MS spectra against genes predicted from all binned metagenomic data sets described above (108,923 entries). The search parameters used were previously described (47). A decoy database searching methodology was used to control the false discovery rate at the unique peptide level to ~0.1% (48). For quantification, peptide reporter ion intensities were captured across all channels and compared by calculating the summed protein intensity values across all fractions. Peptide/protein redundancy was maintained throughout.

### Growth conditions and protein expression.

Genes encoding the *T. bispora* hydrolytic cellulase sequences from enrichment cultures were synthesized by GenScript (Piscataway, NJ). Sequences contain a His tag terminus and were codon optimized for expression in *E. coli*, and the signal peptides were removed. Genes were provided in the entry vector pEOpt3 and were cloned using Golden Gate assembly (49) into *E. coli* DH10B. All reagents were purchased from New England Biolabs (Ipswich, MA). Briefly, the desired gene was digested out of the entry vector, ligated into a new destination vector, and transformed into an *E. coli* expression strain. Digestion was completed by incubating the gene plus entry vector (0.1 µg) with destination vector (50 ng pFil-B), 1× BSA, 1 mM ATP, 1× CutSmart buffer, and BsaI HP restriction enzyme with up to 10 µl of water at 37°C for 1 h. Next, T4 ligase (1 µl) was added to the digested product, and the mixture was incubated with 25 cycles of 37°C for 3 min and 16°C for 4 min, 50°C for 10 min, and 80°C for 10 min and then cooled to 4°C. The product was then transformed into chemically competent *E. coli* DH10B cells for storage and again into chemically competent *E. coli* NEB Express for heterologous expression of proteins. Starter cultures (50 ml) of *E. coli* NEB Express-harboring plasmids were grown overnight in LB medium containing 25 µg/ml kanamycin at 37°C and shaken at 200 rpm in rotary shakers. Expression was performed in Terrific Broth with 2% glycerol, 25 µg/ml kanamycin, and 2 mM MgSO_4_. Starter cultures were used to inoculate 1 liter of expression medium in a 2-liter baffled Erlenmeyer flask and incubated at 18°C while shaking (200 rpm) and induced with 500 µM isopropyl-β-d-1-thiogalactopyranoside (IPTG). Following induction, cultures were again incubated at 18°C. At 22 h, cultures were divided and centrifuged at 15,500 × *g* for 30 min. Cell pellets were resuspended in 25 ml 50 mM HEPES plus 150 mM NaCl plus 20 mM imidazole (pH 7.4) and homogenized with an EmulsiFlex-C3 instrument (Avestein, Inc., Ontario, Canada). Lysates were collected via centrifugation at 75,000 × *g* for 30 min and 0.45-µm-pore filtered to remove large particles prior to purification. Polyhistidine-tagged proteins were purified on Ni-nitrilotriacetic acid (NTA) resin (Thermo Scientific, Rockford, IL) and stored at 4°C until ready for use. The proteins were >90% pure as visualized by SDS-PAGE.

### Saccharification with purified proteins on biomass.

Saccharifications using purified proteins were conducted on 2% (wt/vol) Avicel. Each mixture was prepared in 50 mM MES (pH 6.0) with 40 mg total protein per g glucan in biomass to a final volume of 100 µl in a 96-well microtiter plate. Saccharifications were carried out 60°C in a shaker for 72 h. Samples were harvested and analyzed by HPLC as described above.

### NIMS assay.

Time course reactions were set up by incubating 25 µl enzyme solution of the GH12, GH6_exo, and GH48 proteins (60 mg/g glucan) in a volume of 500 µl 100 mM NaOAc (pH 5.5) and 5 mg Avicel or phosphoric acid-swollen cellulose (PASC) (50). One Whatman no. 1 filter paper disc (GE Healthcare, Pittsburgh, PA), which was produced with a hole puncher and averaged ~3.5 mg, was added to each reaction mixture. The reaction mixtures were incubated at 60°C for 8 h, and 2-µl aliquots were removed at the indicated time points and analyzed by NIMS as previously described with ^13^C-labeled glucose as a standard (24). The processivity measurement was performed with Whatman no. 1 filter paper as previously described (51).

### Accession number(s).

The four metagenomes can be accessed at the JGI IMG website (http://img.jgi.doe.gov/) with IMG genome ID 3300001232 (passage 2A), 3300005157 (passage 3A), 3300000906 (passage 2B), and 3300005137 (passage 3B). The gene sequences and plasmid constructs for the *T. bispora* glycoside hydrolases GH12 (JPUB_007416), GH6_exo (JPUB_007418), GH48 (JPUB_007420), and GH6_endo (JPUB_007422) are available from the public version of the JBEI Registry (https://public-registry.jbei.org) and are physically available from the authors and/or Addgene (http://www.addgene.org) upon request. The mass spectrometry proteomics data have been deposited in the ProteomeXchange Consortium (52) (http://www.proteomexchange.org/) via the PRIDE partner repository with the data set identifier PXD004204.

## SUPPLEMENTAL MATERIAL

Figure S1  Scatterplot of correlation coefficients and *P* values of 117 proteins that are significantly correlated (*P* ≤ 0.05) with the produced glucose amounts of the four samples. Point sizes indicate proteomic abundances in lineage A passage 2; GH genes are colored red. Download Figure S1, PDF file, 0.1 MB

Figure S2  Production of glucose from Avicel by *T. bispora* cellulases expressed in *E. coli*. Details of the experiment are described in the legend to Fig. 4. Download Figure S2, PDF file, 0.02 MB

Figure S3  Time course experiments for GH6_exo protein hydrolysis of Avicel (A), filter paper (B), and PASC (C). Sugar products were derivatized with oxime and measured by NIMS. Each point represents the average from three independent replicates. Download Figure S3, PDF file, 0.1 MB

Figure S4  Time course experiments for GH48 protein hydrolysis of Avicel (A), filter paper (B), and PASC (C). Sugar products were derivatized with oxime and measured by NIMS. Each point represents the average from three independent replicates. Download Figure S4, PDF file, 0.1 MB

Table S1  Summary of individual genomes recovered from the metagenomes. Table S1, PDF file, 0.04 MB

Table S2  Relative abundances and measured coverages of individual genomes measured from metagenomes. Table S2, PDF file, 0.1 MB

Table S3  Proteomic abundances of the recovered individual genomes. Table S3, PDF file, 0.1 MB

Table S4  Proteomic abundances of glycoside hydrolases (GH) from the recovered genomes. Table S4, PDF file, 0.1 MB

Table S5  Preason’s correlation coefficients and *P* values between the measured proteomic abundances and produced glucose amounts of the four samples. Table S5, PDF file, 0.03 MB
