# Supplementary figures and images for: Comparative Community Proteomics Demonstrates the Unexpected Importance of Actinobacterial Glycoside Hydrolase Family 12 Protein for Crystalline Cellulose Hydrolysis
Source: mBio. 2016 Aug 23;7(4):e01106-16. doi: 10.1128/mBio.01106-16 (PMC4999548; doi:10.1128/mBio.01106-16)

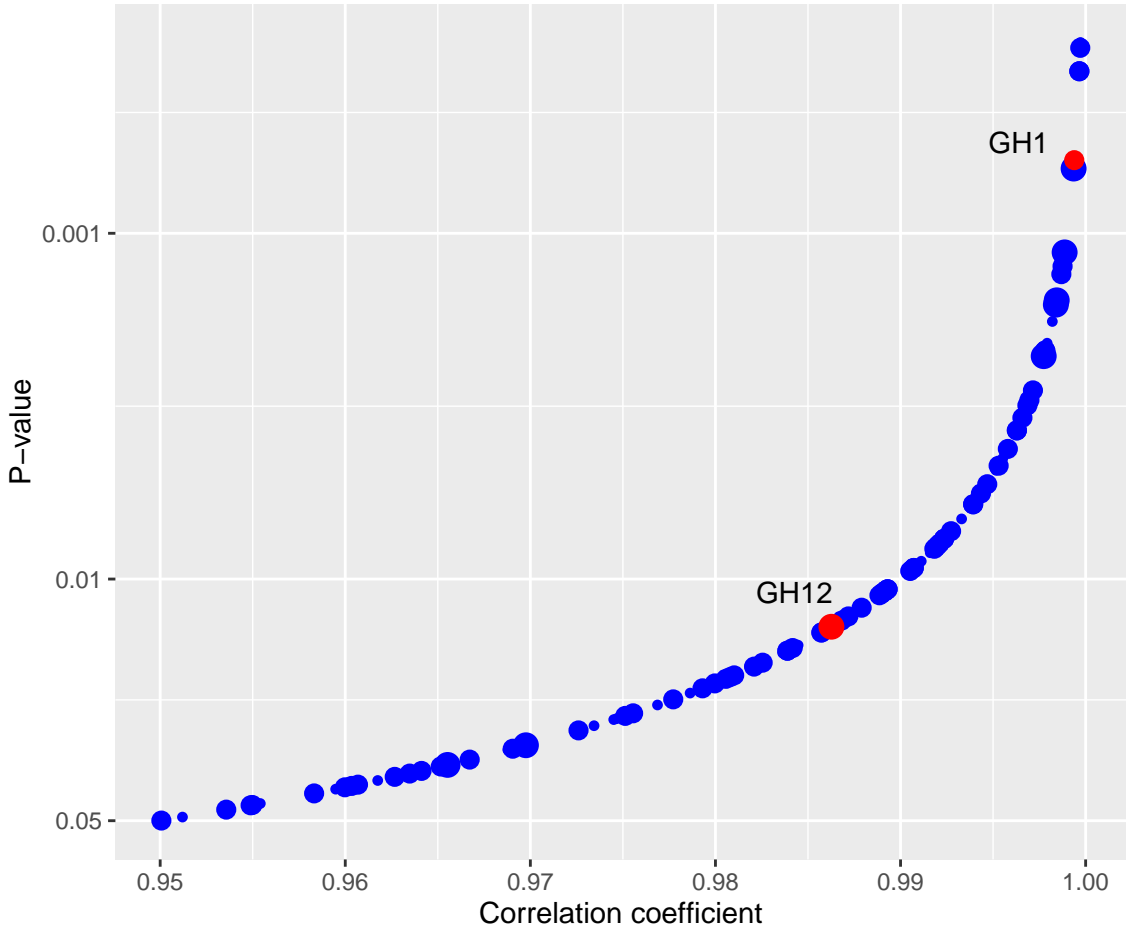

GH genes

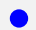

Non-GH

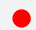

GH

Protein abundances in 60A Passage 2

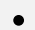

1000000

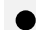

10000000

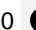

100000000

Supplement: Figure S1 — Scatterplot of correlation coefficients and P values of 117 proteins that are significantly correlated (P ≤ 0.05) with the produced glucose amounts of the four samples. Point sizes indicate proteomic abundances in lineage A passage 2; GH genes are colored red. Download [file mbo004162951sf1.pdf]

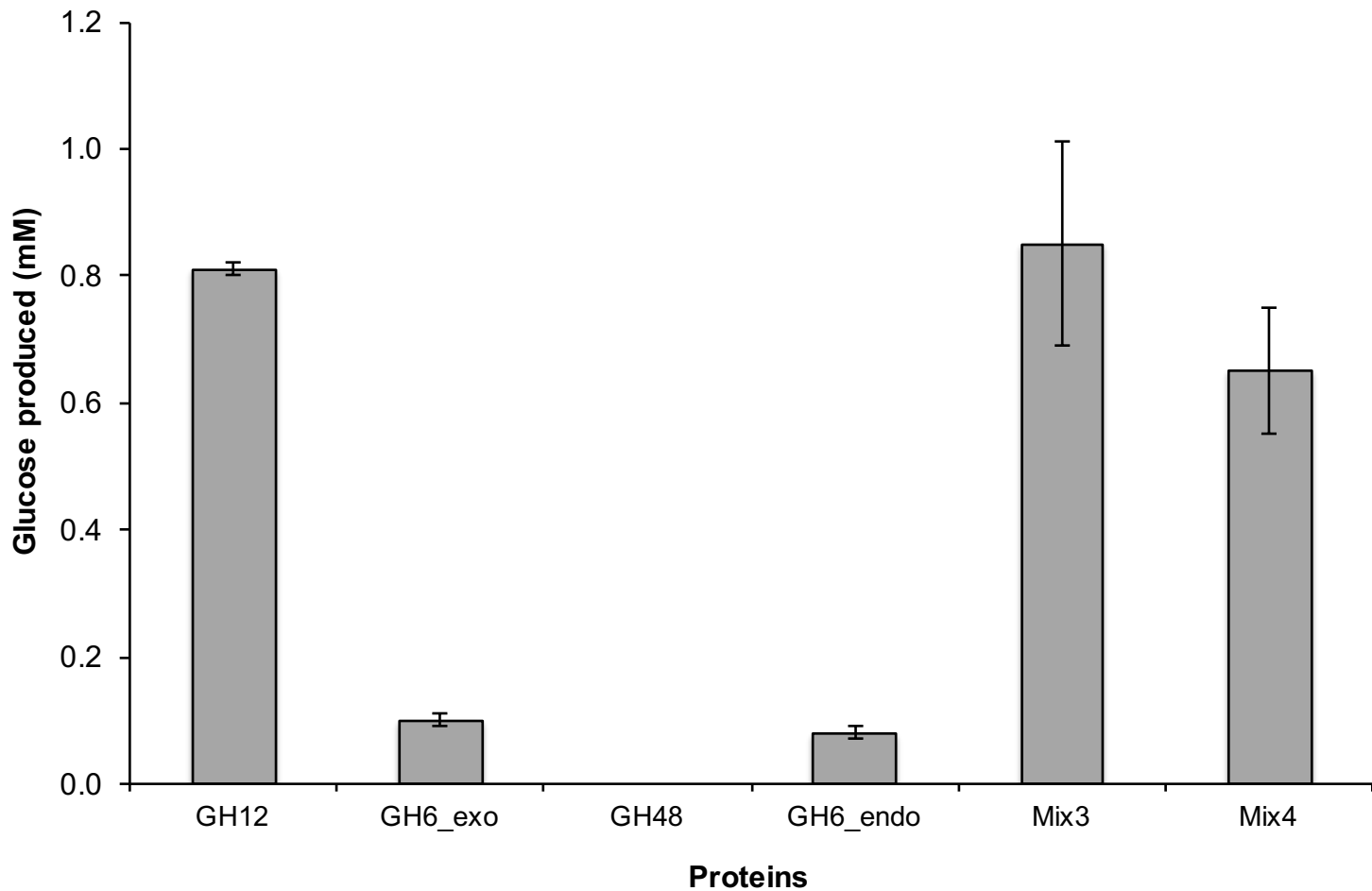

Supplement: Figure S2 — Production of glucose from Avicel by T. bispora cellulases expressed in E. coli. Details of the experiment are described in the legend to Fig. 4. Download [file mbo004162951sf2.pdf]

A.

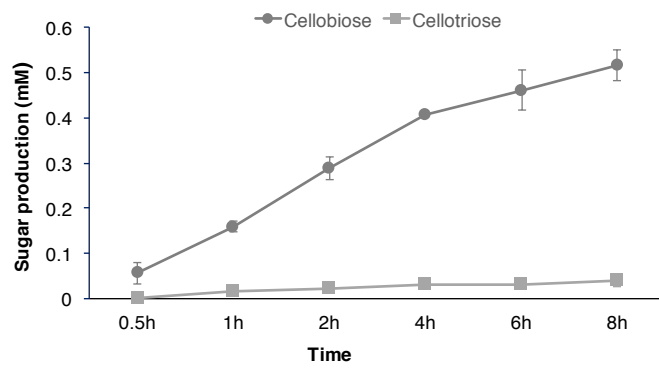

B.

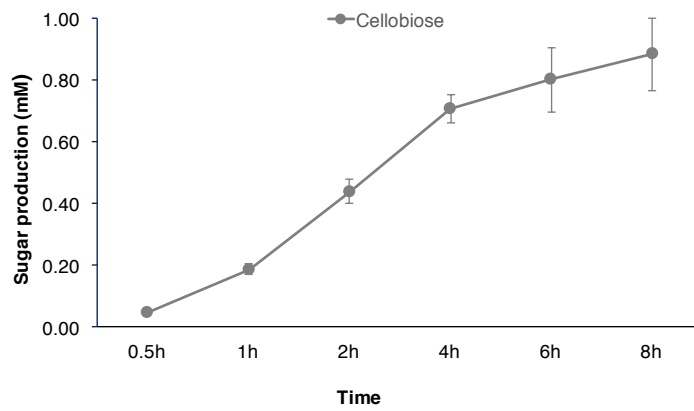

C.

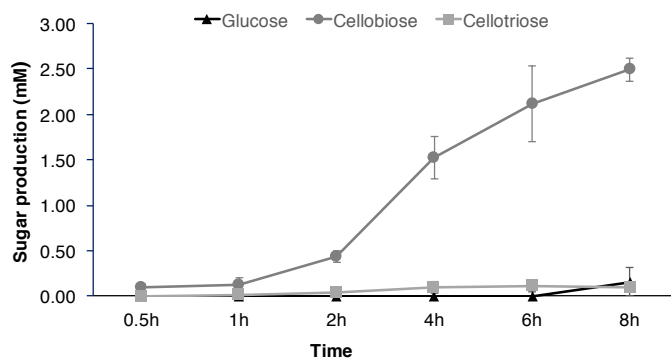

Supplement: Figure S3 — Time course experiments for GH6_exo protein hydrolysis of Avicel (A), filter paper (B), and PASC (C). Sugar products were derivatized with oxime and measured by NIMS. Each point represents the average from three independent replicates. Download [file mbo004162951sf3.pdf]

**A.**

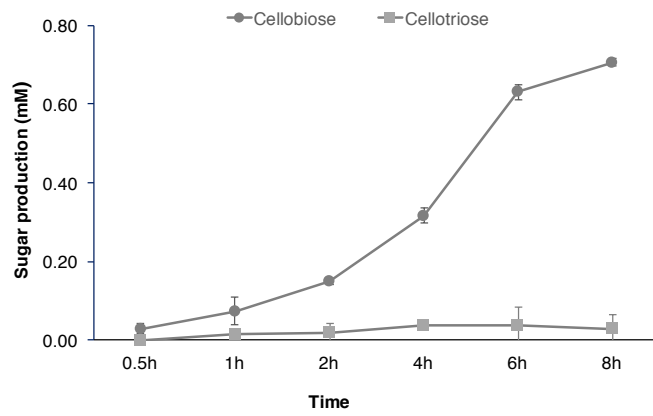

**B.**

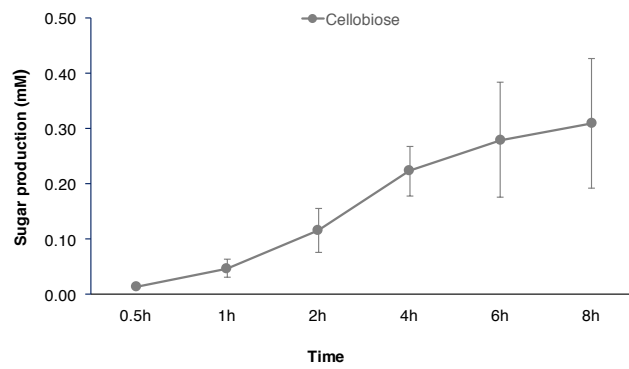

**C.**

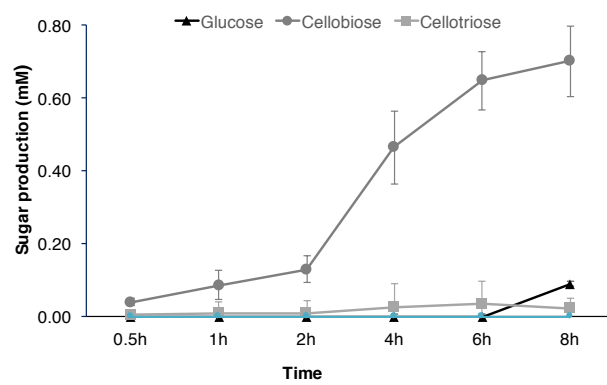

Supplement: Figure S4 — Time course experiments for GH48 protein hydrolysis of Avicel (A), filter paper (B), and PASC (C). Sugar products were derivatized with oxime and measured by NIMS. Each point represents the average from three independent replicates. Download [file mbo004162951sf4.pdf]
