# Supplementary material for: Comparative Community Proteomics Demonstrates the Unexpected Importance of Actinobacterial Glycoside Hydrolase Family 12 Protein for Crystalline Cellulose Hydrolysis
Source: mBio. 2016 Aug 23;7(4):e01106-16. doi: 10.1128/mBio.01106-16 (PMC4999548; doi:10.1128/mBio.01106-16)
Supplement: Table S1 — Summary of individual genomes recovered from the metagenomes. [file mbo004162951st1.pdf]

Bin	Phylum	Taxonomy	Completeness	Genome size	GC%
1	Actinobacteria	*Thermobispora bispora*	90.7%	3818041	71.95
2	Bacteroidetes	Chitinophagaceae	95.3%	2844373	46.13
3	Firmicutes	Paenibacillaceae	99.1%	3243081	62.58
4	Firmicutes	*Thermobacillus composti*	96.3%	4054056	61.04
5	Chloroflexi	*Sphaerobacter thermophilus*	93.5%	4064388	68.22
6	Firmicutes	*Caldibacillus debilis*	98.1%	3187021	51.22
7	Proteobacteria	Betaproteobacteria	96.3%	2781671	60.19
8	Firmicutes	*Thermobacillus* sp.	80.4%	3118454	64.58
9	Firmicutes	*Thermobacillus* sp.	81.3%	4214511	62.43
10	Actinobacteria	*Thermocrispum agreste*	99.1%	4555205	69.75
11	Proteobacteria	Betaproteobacteria	99.1%	2963958	66.12
12	Firmicutes	*Thermobacillus* sp.	33.6%	1968828	64.96
13	Proteobacteria	Rhizobiales	49.5%	2408050	68.96
14	Firmicutes	Paenibacillaceae	99.1%	4167122	54.99
15	Actinobacteria	Streptosporangiaceae	99.1%	5370526	73.23
16	Proteobacteria	Xanthomonadaceae	53.3%	3192096	65.4
17	Proteobacteria	Xanthomonadaceae	84.1%	4705527	70.83
18	Deinococcus-Thermus	*Thermus thermophilus*	95.3%	2598646	68.8
19	Firmicutes	Paenibacillaceae	88.8%	3462697	61.49
20	Firmicutes	*Geobacillus thermodenitrificans*	88.8%	2672194	50.33
21	Proteobacteria	Alphaproteobacteria	89.7%	3562680	69.03
22	Firmicutes	Bacillaceae	99.1%	6605220	39.97
23	Thermobaculum	*Thermobaculum terrenum*	93.5%	3716369	53.97
24	Chloroflexi	Thermomicrobia	95.3%	5058975	63.83
25	Firmicutes	Paenibacillaceae	97.2%	3787746	54.1
26	Deinococcus-Thermus	Deinococci	92.5%	3262311	72.85
27	Firmicutes	Paenibacillaceae	100.0%	4629858	58.08
28	Bacteroidetes	*Rhodothermus marinus*	100.0%	3415689	64.17
29	Proteobacteria	Myxococcales	98.1%	3044921	69.76
30	Firmicutes	Bacillales	97.2%	2871974	60.25
