# Supplementary material for: Comparative Community Proteomics Demonstrates the Unexpected Importance of Actinobacterial Glycoside Hydrolase Family 12 Protein for Crystalline Cellulose Hydrolysis
Source: mBio. 2016 Aug 23;7(4):e01106-16. doi: 10.1128/mBio.01106-16 (PMC4999548; doi:10.1128/mBio.01106-16)
Supplement: Table S2 — Relative abundances and measured coverages of individual genomes measured from metagenomes. [file mbo004162951st2.pdf]

	Relative Abundances (%)				Absolute Coverages[1]			
Bin	60A Pas2	60A Pas3	60B Pas2	60B Pas3	60A Pas2	60A Pas3	60B Pas2	60B Pas3
1	81.7%	89.2%	7.8%	22.0%	1842.38	3796.75	220.23	527.82
2	6.1%	0.2%	0.0%	1.8%	136.53	6.66	0.36	44.04
3	3.3%	0.2%	0.2%	0.1%	73.75	8.79	5.2	2.3
4	2.2%	1.6%	3.4%	40.3%	48.96	66.51	95.32	964.28
5	1.7%	1.0%	1.3%	0.5%	38.2	42.1	35.55	13.1
6	1.1%	1.4%	2.4%	10.0%	24.07	58.01	66.99	238.96
7	0.8%	0.5%	1.0%	7.0%	18.12	21.81	28.49	168.2
8	0.7%	0.0%	0.1%	0.0%	15.61	0.13	3.12	0.21
9	0.6%	0.2%	0.8%	0.5%	13.75	10.3	23.69	11.51
10	0.4%	4.4%	0.0%	0.0%	8.14	188.09	0.67	0.35
11	0.4%	0.0%	0.4%	0.1%	8.36	0.85	10.23	1.83
12	0.0%	0.0%	0.4%	0.0%	0.42	0.08	11.69	0.03
13	0.2%	0.1%	0.0%	0.0%	3.48	2.28	0.18	0.43
14	0.1%	0.1%	0.0%	0.2%	2.95	3.13	1.36	5.32
15	0.1%	0.4%	0.1%	1.1%	2.89	15.92	2.65	26.8
16	0.1%	0.0%	0.1%	0.0%	2.48	1.1	1.55	0.95
17	0.1%	0.0%	0.1%	0.2%	2.08	0.49	2.45	3.64
18	0.1%	0.0%	1.8%	5.4%	1.9	0.66	49.64	130.08
19	0.1%	0.0%	14.7%	0.1%	1.7	0.2	417.27	1.54
20	0.1%	0.3%	0.0%	0.6%	1.69	11.21	0.62	15.43
21	0.1%	0.0%	0.1%	0.1%	1.34	0.13	4.18	1.77
22	0.1%	0.3%	0.0%	0.4%	1.18	11.08	1.13	9.14
23	0.1%	0.2%	0.0%	0.0%	1.14	9.22	1.09	0.1
24	0.0%	0.0%	0.1%	1.1%	1.09	0.14	1.61	26.31
25	0.0%	0.0%	0.3%	0.4%	0.76	0.53	7.73	9.27
26	0.0%	0.0%	1.3%	0.2%	0.48	0.13	38.11	4.05
27	0.0%	0.0%	0.1%	0.6%	0.27	0.6	4.23	13.32
28	0.0%	0.0%	61.3%	3.1%	0.13	1.21	1738.09	74.72
29	0.0%	0.0%	2.1%	0.1%	0.07	0.07	60.64	3.49
30	0.0%	0.0%	0.0%	4.0%	0.01	0.01	0.03	94.86

[1] Measured by MaxBin 2.0
