# Supplementary material for: Comparative Community Proteomics Demonstrates the Unexpected Importance of Actinobacterial Glycoside Hydrolase Family 12 Protein for Crystalline Cellulose Hydrolysis
Source: mBio. 2016 Aug 23;7(4):e01106-16. doi: 10.1128/mBio.01106-16 (PMC4999548; doi:10.1128/mBio.01106-16)
Supplement: Table S3 — Proteomic abundances of the recovered individual genomes. [file mbo004162951st3.pdf]

Bin	Relative Proteomic Abundance (%)				Measured Proteomic Abundances			
	60A Pas2	60A Pas3	60B Pas2	60B Pas3	60A Pas2	60A Pas3	60B Pas2	60B Pas3
1	49.4%	33.1%	10.7%	10.0%	2098004621	1623416496	279026746.4	292384867
2	0.6%	2.6%	0.8%	0.5%	23394622.01	126191377.6	21787316.26	14016073
3	2.1%	23.2%	3.2%	2.2%	90433730.07	1135875852	84677818.94	63564447
4	6.4%	6.1%	9.7%	13.5%	269944320.5	297842523.1	252019783.9	393526274
5	2.5%	2.0%	2.8%	2.4%	106478042.2	98147705.76	73532173.04	70201773
6	10.8%	6.0%	12.3%	18.3%	460523987.9	294334273	319622539.5	533651899
7	2.9%	1.7%	3.0%	6.8%	124683325.5	84051234.08	78047382.58	199332903
8	1.7%	1.8%	3.3%	4.0%	72009641.03	90519753.47	85184381.05	115903969
9	1.7%	2.0%	5.3%	3.7%	70535625.29	96409234.09	138857360.5	108537181
10	1.0%	0.4%	0.5%	0.7%	41114492.37	20050819.49	13923531.3	19543450
11	0.2%	0.2%	0.5%	0.6%	9248516.676	9814355.707	12559656.33	18018821
12	0.7%	0.6%	1.4%	2.1%	31269130.42	29523532.26	35717301.91	61533663
13	0.1%	0.1%	0.1%	0.3%	5139360.324	6018601.396	3279057.991	8937519
14	1.4%	1.7%	2.5%	3.2%	59149943.12	82864928.94	66364539.57	93116153
15	2.5%	1.8%	2.0%	2.6%	104199876.9	85987175.82	53127718.68	76496703
16	0.3%	0.2%	0.6%	0.4%	11712150.3	7452062.323	14566596.66	12599807
17	0.2%	0.2%	0.3%	1.1%	10315246.52	9824916.572	6984268.298	32172658
18	1.0%	1.3%	1.4%	2.7%	44257568.48	63221515.37	37821457.74	77571108
19	1.8%	2.2%	16.1%	3.7%	74596075.13	109386876.3	419820427.2	108576969
20	1.8%	1.1%	1.8%	3.8%	76857498.24	55853703.39	45835288.42	109698388
21	0.2%	0.2%	0.4%	0.5%	8620738.196	9158657.951	11605899.13	15976931
22	1.7%	1.2%	2.1%	3.6%	71547674.38	57231678.8	54948647.17	105090852
23	0.4%	0.6%	0.4%	0.6%	19110307.19	28382171.48	9970329.827	16012609
24	0.8%	1.0%	1.1%	1.4%	36101579.07	47310465.03	27446468.35	39900893
25	1.1%	1.2%	2.2%	2.2%	44929524.01	58869455.69	58354150.75	63372235
26	0.6%	0.6%	0.8%	0.7%	27162759.9	30865824.25	20717461.38	20748277
27	1.6%	1.9%	2.6%	2.8%	65930386.86	92824379.18	67547555.19	82475463
28	3.0%	3.6%	8.7%	2.2%	128926222	174543548.5	226177408.3	63004626
29	0.2%	0.2%	1.0%	0.5%	9230129.637	12131962.25	27277293.15	13106401
30	1.2%	1.4%	2.4%	2.8%	52895011.84	67649917.36	61916535.7	81660921
