# Supplementary material for: Comparative Community Proteomics Demonstrates the Unexpected Importance of Actinobacterial Glycoside Hydrolase Family 12 Protein for Crystalline Cellulose Hydrolysis
Source: mBio. 2016 Aug 23;7(4):e01106-16. doi: 10.1128/mBio.01106-16 (PMC4999548; doi:10.1128/mBio.01106-16)
Supplement: Table S4 — Proteomic abundances of glycoside hydrolases (GH) from the recovered genomes. [file mbo004162951st4.pdf]

Bin	Relative GH Proteomic Abundance (%)				Measured GH Proteomic Abundances			
	60A Pas2	60A Pas3	60B Pas2	60B Pas3	60A Pas2	60A Pas3	60B Pas2	60B Pas3
1	76.3%	82.4%	37.2%	16.4%	245916691.6	417779375.9	24438397	10060451
2	0.2%	0.6%	0.8%	0.6%	510313.8	2859002	525543.9	370469
3	0.1%	0.7%	0.8%	0.6%	348428.8	3362229	508396.4	369553
4	1.6%	1.8%	10.3%	17.1%	5163571	8931808	6791344	10512541
5	0.3%	0.3%	1.8%	1.3%	989252.4	1378208	1160039	810277
6	1.4%	0.5%	5.2%	7.1%	4444777	2600173	3385301	4339170
7	0.1%	0.0%	0.3%	0.5%	284668.2	231464.2	204548.8	296554
8	0.7%	0.5%	7.3%	13.2%	2216946	2526624	4820928	8087641
9	0.3%	0.4%	3.6%	4.9%	867449.7	2099445	2338448	2980132
10	0.1%	0.1%	0.2%	0.2%	359574.7	272604.9	139069.2	138194
11	0.0%	0.0%	0.0%	0.0%	0	0	0	0
12	0.1%	0.1%	0.6%	1.2%	322568.4	695651.9	384710.8	754699
13	0.0%	0.0%	0.0%	0.0%	0	0	0	0
14	0.2%	0.1%	2.7%	3.9%	555342.9	700581.7	1798328	2417132
15	0.0%	0.0%	0.2%	0.3%	150993.5	189665.5	129102.6	190243
16	0.0%	0.0%	0.0%	0.0%	0	0	0	0
17	0.0%	0.0%	0.1%	0.2%	31476.54	26115.68	36862.65	100231
18	3.9%	2.5%	0.8%	1.5%	12540212	12561166	504152.5	917044
19	0.3%	0.4%	4.9%	4.9%	1034027	2139665	3227224	3037737
20	0.4%	0.1%	2.8%	4.6%	1201154	739288.9	1828719	2803573
21	0.0%	0.0%	0.2%	0.0%	12578.27	3799.989	122450.7	12147
22	0.4%	0.2%	4.2%	4.9%	1172598	762962.4	2778926	3032178
23	0.1%	0.0%	0.1%	0.1%	173023.4	82728.62	67844.58	40073
24	0.1%	0.1%	0.6%	0.8%	280453.6	472607	370064	517119
25	0.0%	0.0%	0.3%	3.1%	129289.7	191390.6	199061.4	1925644
26	0.0%	0.0%	0.0%	0.0%	0	0	0	0
27	0.1%	0.1%	2.5%	3.5%	377283.8	492994.4	1654755	2122634
28	13.0%	8.7%	8.9%	3.8%	41803754	44146465	5849925	2322803
29	0.0%	0.0%	0.0%	0.0%	0	0	0	0
30	0.4%	0.4%	3.7%	5.3%	1299722	1937619	2433091	3286095
Sum	100%	100%	100%	100%	322269458.7	507404259.8	65697273.53	61444334
