# Supplementary material for: Comparative Community Proteomics Demonstrates the Unexpected Importance of Actinobacterial Glycoside Hydrolase Family 12 Protein for Crystalline Cellulose Hydrolysis
Source: mBio. 2016 Aug 23;7(4):e01106-16. doi: 10.1128/mBio.01106-16 (PMC4999548; doi:10.1128/mBio.01106-16)
Supplement: Table S5 — Preason’s correlation coefficients and P values between the measured proteomic abundances and produced glucose amounts of the four samples. [file mbo004162951st5.pdf]

Gene	Pearson's correlation coefficient	p-value
GH6_exo	0.79	0.2
GH12	0.99	0.01
AA10	0.3	0.69
GH6_endo	0.18	0.81
GH48	0.44	0.55
